# Pulmonary aspergillosis in US Veterans with COVID-19: a nationwide, retrospective cohort study

**DOI:** 10.1017/ash.2024.476

**Published:** 2025-01-30

**Authors:** Kaitlin Swinnerton, Nathanael R. Fillmore, Ikwo Oboho, Janet Grubber, Mary Brophy, Nhan V Do, Paul A Monach, Westyn Branch-Elliman

**Affiliations:** 1 VA Boston Cooperative Studies Program, Boston, MA, USA; 2 Dana Farber Cancer Institute, Boston, MA, USA; 3 Harvard Medical School, Boston, MA, USA; 4 VA Boston Healthcare System, Boston, MA, USA; 5 VA North Texas Health Care System, Dallas, TX, USA; 6 UT Southwestern School of Medicine, Dallas, TX, USA; 7 Boston University School of Medicine, Boston, MA, USA; 8 Greater Los Angeles VA Healthcare System, Department of Medicine, Los Angeles, CA, USA; 9 UCLA David Geffen School of Medicine, Los Angeles, CA, USA; 10 VA Center for the Study of Healthcare Innovation, Implementation, and Policy, Los Angeles, CA, USA

## Abstract

**Background:**

COVID-associated pulmonary aspergillosis (CAPA) was described early in the pandemic as a complication of SARS-CoV-2. Data about incidence of aspergillosis and characteristics of affected patients after mid-2021 are limited.

**Methods:**

A retrospective, nationwide cohort of US Veterans with SARS-CoV-2 from 1/1/2020 to 2/7/2024 was created. Potential cases of aspergillosis ≤12 weeks of a SARS-CoV-2 test were flagged electronically (based on testing results indicative of invasive fungal infection, antifungal therapy, and/or ICD-10 codes), followed by manual review to establish the clinical diagnosis of pulmonary aspergillosis. Incidence rates were calculated per 10,000 SARS-CoV-2 cases. Selected clinical characteristics included age >70, receipt of immune-compromising drugs, hematologic malignancy, chronic respiratory disease, vaccination status, and vaccine era. Multivariate logistic regression was used to estimate the independent effects of these variables via adjusted odds ratios (aOR).

**Results:**

Among 674,343 Veterans with SARS-CoV-2, 165 were electronically flagged for review. Of these, 66 were judged to be cases of aspergillosis. Incidence proportions ranged from 0.30/10,000 among patients with zero risk factors to 34/10,000 among those with ≥3 risk factors; rates were similar in the pre- and post-vaccination eras. The 90-day mortality among aspergillosis cases was 50%. In the multivariate analysis, immune suppression (aOR 6.47, CI 3.84–10.92), chronic respiratory disease (aOR 3.57, CI 2.10–6.14), and age >70 (aOR 2.78, CI 1.64–4.80) were associated with aspergillosis.

**Conclusions:**

Patients with underlying risk factors for invasive aspergillosis continue to be at some risk despite SARS-CoV-2 immunization. Risk in patients without immune suppression or preexisting lung disease is very low.

## Background

COVID-associated pulmonary aspergillosis (CAPA) was described early in the COVID-19 pandemic,^1^ and consensus definitions of the disease were published in 2021.^2^ Intensive care unit (ICU) cohort studies suggested incidence rates ranging from 2 to 33 per 100 patients with COVID-19, although autopsy studies suggested much lower rates (≤2%).^3,4^ In 2020, mortality rates for CAPA were reported to approach or exceed 50%.^3,5^

Multiple prior studies evaluated the epidemiology of and risk factors for CAPA.^2^ Giola *et al* conducted a systematic review of risk factors among studies published through 2023 (N = 1,398 cases of CAPA) and reported nine risk factors: chronic liver disease, hematologic malignancies, chronic obstructive pulmonary disease (COPD), cerebrovascular disease, diabetes mellitus, use of mechanical ventilation, renal replacement therapy, and receipt of corticosteroids and/or IL-6 inhibitors to treat severe COVID-19.^6^ Other studies suggest that receipt of the corticosteroid dexamethasone as part of treatment for COVID-19 may increase CAPA risk.^7,8^ Limited data from these studies are available regarding traditional risk factors for pulmonary aspergillosis, including exposure to immunosuppressive medications before infection, such as immunomodulatory drugs or chemotherapeutic agents; however, the consensus panel noted “the absence of classic host factors for invasive fungal infections” among identified CAPA cases, suggesting substantial risk in the general population, rather than concentrated risk among patients with immune suppression.(2).^9,10^

Since the early reports of CAPA, advancements in treatment and prevention of COVID-19 have changed SARS-CoV-2 disease severity.^11,12^ Despite these improvements, some patients remain susceptible to severe disease^13–15^ despite vaccination or use of antiviral drugs.^13^ This same population of patients with immune suppression and chronic lung disease is also the population at highest risk of pulmonary aspergillosis in the absence of SARS-CoV-2 infection.^16^ Thus, the aim of this study was to measure the incidence of clinically significant pulmonary aspergillosis in patients with recent SARS-CoV-2 infection before and after availability of SARS-CoV-2 vaccinations and to describe risk factors for aspergillosis. Because the consensus definition of CAPA requires bronchoalveolar lavage (BAL) or histopathology for definite cases,^2^ which are used less frequently in the US than in Europe and which are known to be highly specific but insensitive,^17,18^ we used a clinical definition based on physician review of cases, including microbiologic results and the reasoning expressed by the treating physicians (specialists in infectious diseases or pulmonology) who interpreted results in the context of clinical status and imaging; this approach is aligned with other studies that aimed to estimate prevalence.^17^ Our primary hypothesis was that the incidence of aspergillosis would be decreased in the post-vaccination era. A secondary hypothesis was that the major ongoing risk factor for aspergillosis would be immune suppression.

## Methods

### Data sources, participants, and study design

Data were obtained from the VA COVID-19 Shared Data Resource and the Corporate Data Warehouse (CDW), which collates electronic health record (EHR) data from Veterans Affairs (VA) facilities nationwide. We included Veteran patients who developed SARS-CoV-2 infection between January 1, 2020, and February 7, 2024. Only the first laboratory-confirmed SARS-CoV-2 infection (polymerase chain reaction or antigen test) was used for each patient, since distinguishing a new infection from residual virus would be challenging, particularly among immune-suppressed patients.

### Outcomes and predictors

Chart review was used to define cases of clinically significant pulmonary aspergillosis. A simple electronic algorithm consisting of 3 variables was used to flag cases for manual review: i) receipt of at least one medication used to treat invasive pulmonary aspergillosis (amphotericin, ambisome, voriconazole, posaconazole, isavuconazole, caspofungin, micafungin, and aniodulafungin) and either ii) at least one use of an ICD10 code for invasive pulmonary aspergillosis (B44) or iii) microbiologic support (a positive culture for aspergillus from BAL or sputum, or a positive aspergillus galactomannan antigen test in BAL fluid or serum, within 12 weeks after the positive test for SARS-CoV-2 (polymerase chain reaction or antigen). The requirement for only 2 of these 3 variables was made to improve sensitivity in a setting in which manual review was feasible. Each putative case was reviewed by at least one physician (W.B.-E. or P.A.M.), who then asked the other to also review the case in the event of uncertainty how to classify; ongoing uncertainty was resolved through discussion. The reviewers took into account microbiologic testing and notes written by the specialists in infectious diseases or pulmonology, who interpreted those results in the context of a patient’s clinical course, imaging, and risk factors for aspergillosis. The reviewers did not compile details of imaging nor negative results of BAL or microbiologic testing among cases classified as aspergillosis, nor the reasons for not classifying other cases as aspergillosis. Examples of reasons why a case might be captured by the electronic screen but not be classified as post-SARS-CoV-2 aspergillosis included: ICD-10 code plus use of an antifungal drug for prophylaxis rather than treatment, ICD-10 code plus use of an antifungal drug for aspergillosis preceding the SARS-CoV-2 infection (eg, from known prior aspergillus infection or allergic bronchopulmonary aspergillosis), or a positive non-specific serum marker without confirmatory imaging, microbiology, or clinical diagnosis.

Clinical and demographic variables were chosen, among patients with and without aspergillosis following SARS-CoV-2 infection, based on prior literature about severe COVID-19 and/or CAPA. These included vaccination status (fully vaccinated or not fully vaccinated, defined as completion of an initial vaccination series), vaccine era (vaccine era = infection occurring after January 16, 2021), age > 70, treatment with immune-suppressive drugs (yes vs no for: chemotherapy, cytokine blockers, glucocorticoids, leukocyte inhibitors, and/or lymphocyte depleting drugs) *before* SARS-CoV-2 infection, and selected comorbidities. We did not include all comorbidities previously associated with risk of CAPA but focused on those associated with an immune-suppressed state (history of hematologic malignancy or organ transplantation) or history of respiratory disease (COPD, bronchiectasis, interstitial lung disease, or asthma) (see Supplementary Table 1 for all definitions). We identified use of dexamethasone after SARS-CoV-2 infection in patients with aspergillosis, since it has been proposed as a risk factor for CAPA. However, we did not include dexamethasone in multivariate modeling, since it is not possible to distinguish an association due to immune-suppression versus it being a surrogate marker of severe COVID-19. Demographic data were also obtained for patients with and without aspergillosis. Data about mechanical ventilation and ICU admission or high-flow oxygen supplementation were also manually extracted for patients with aspergillosis.

### Statistical analysis

We compared the incidence proportion of clinically significant pulmonary aspergillosis in the pre-vaccine vs vaccine eras and in those who received a complete initial vaccination series (eg, fully vaccinated) versus not fully vaccinated. We also compared the distribution of pre-specified risk factors among Veterans who developed aspergillosis within 12 weeks of SARS-CoV-2 infection vs Veterans who did not develop aspergillosis within that time frame. We then fit a multivariate logistic regression model to estimate the odds of aspergillosis within 12 weeks following SARS-CoV-2 diagnosis among patients based on their baseline covariates. *A priori* we selected a subset of the established risk factors for severe COVID-19 and/or CAPA (vaccine era, fully vaccinated status, age > 70, prior treatment with immune-compromising drugs, history of hematologic malignancy, and history of chronic respiratory disease) and included each as an independent variable in the multivariate model, with results expressed as adjusted odds ratios (aOR) and their 95% confidence intervals (CIs). Logistic regression was used to enhance the value of an essentially descriptive study rather than as an effort to rigorously estimate the contributions of a large number of variables.

This study was approved by the VA Boston Research and Development Committee prior to data collection and analysis.

## Results

During the period from 1/1/2020-2/7/2024, 674,343 Veteran patients with SARS-CoV-2 infection were identified. The median age of the cohort was 59; 77.6% were male, and 43.2% completed at least the primary vaccination series. Among these 674,343 infections, 165 cases of possible clinically significant pulmonary aspergillosis were flagged by the electronic algorithm. Among these 165 cases, 66 were classified as true aspergillosis cases following chart review (incidence proportion 1.0 cases of aspergillosis per 10,000 SARS-CoV-2 infections). Demographic and clinical characteristics of patients with and without aspergillosis are shown in Table 1. Among the 66 cases of aspergillosis, all had use of an ICD-10 code and an antifungal drug, and 64 had details on microbiologic testing available in the VA system. Of these 64 cases, 36 were supported by sputum culture alone, 10 by BAL culture alone, 7 by galactomannan testing (BAL or serum) alone, 3 by tissue pathology alone, and 8 by galactomannan testing plus culture or pathology. The great majority (63/66, 95.5%) were hospitalized. Severe respiratory illness was apparent in most cases, with 54/66 (81.8%) patients requiring care in the ICU or use of high-flow oxygen and 38/66 (57.6%) requiring mechanical ventilation. Dexamethasone was received by most patients (50/66, 75.8%).

**Table 1.** Characteristics of patients with SARS-CoV-2 infection who did or did not develop clinically-significant pulmonary aspergillosis from March 2020 through March 2024, and results of multivariate logistic regression—US Department of Veterans Affairs

	CSPA	No CSPA	All SARS-CoV-2 cases	Multivariate odds ratio (95% CI)
	*(N = 66)*	*(N = 674277)*	*(N = 674343)*	
Vaccine era	50 (75.8%)	550031 (81.6%)	550081 (81.6%)	0.96 [0.51, 1.85]
Fully vaccinated	27 (40.9%)	291354 (43.2%)	291381 (43.2%)	0.5 [0.28, 0.9]*
Age (Median [25th percentile, 75th percentile])	71.8 [67.0, 74.8]	58.8 [43.7, 71.6]	58.8 [43.7, 71.6]	
Age > 70	41 (62.1%)	190095 (28.2%)	190136 (28.2%)	2.78 [1.64, 4.8]*
Gender				
Male	61 (92.4%)	523544 (77.6%)	523605 (77.6%)	
Female	5 (7.6%)	150731 (22.4%)	150736 (22.4%)	
Non-binary	0 (0%)	2 (0.0%)	2 (0.0%)	
Race				
White	56 (84.8%)	371661 (55.1%)	371717 (55.1%)	
Black or African American	5 (7.6%)	138792 (20.6%)	138797 (20.6%)	
Asian	0 (0%)	7978 (1.2%)	7978 (1.2%)	
American Indian or Alaska Native	1 (1.5%)	4889 (0.7%)	4890 (0.7%)	
Native Hawaiian or Other Pacific Islander	1 (1.5%)	5943 (0.9%)	5944 (0.9%)	
Unknown	3 (4.5%)	145014 (21.5%)	145017 (21.5%)	
Ethnicity				
Hispanic or Latino	2 (3.0%)	61129 (9.1%)	61131 (9.1%)	
Not Hispanic or Latino	61 (92.4%)	490119 (72.7%)	490180 (72.7%)	
Unknown	3 (4.5%)	123029 (18.2%)	123032 (18.2%)	
Region				
Continental	13 (19.7%)	109405 (16.2%)	109418 (16.2%)	
Midwest	18 (27.3%)	147092 (21.8%)	147110 (21.8%)	
North Atlantic	6 (9.1%)	150854 (22.4%)	150860 (22.4%)	
Pacific	17 (25.8%)	122684 (18.2%)	122701 (18.2%)	
Southeast	12 (18.2%)	144241 (21.4%)	144253 (21.4%)	
Missing	0 (0%)	1 (0.0%)	1 (0.0%)	
Selected Risk Factors for Invasive Aspergillosis				
Prior treatment with immunocompromising drugs	33 (50.0%)	55600 (8.2%)	55633 (8.3%)	6.47 [3.84, 10.92]*
History of hematologic malignancy	5 (7.6%)	11461 (1.7%)	11466 (1.7%)	1.9 [0.66, 4.37]
History of chronic respiratory disease	37 (56.1%)	101148 (15.0%)	101185 (15.0%)	3.57 [2.1, 6.14]*
History of transplant	8 (12.1%)	3594 (0.5%)	3602 (0.5%)	
Number of Selected Risk Factors for Invasive Aspergillosis				
0	14 (21.2%)	532382 (79.0%)	532396 (79.0%)	
1	27 (40.9%)	113784 (16.9%)	113811 (16.9%)	
2	19 (28.8%)	26362 (3.9%)	26381 (3.9%)	
3	6 (9.1%)	1701 (0.3%)	1707 (0.3%)	
4	0 (0%)	48 (0.0%)	48 (0.0%)	
**Mortality**				
Died within 90 days after first positive SARS-CoV-2 test	33 (50.0%)	19943 (3.0%)	19976 (3.0%)	
Died within 6 months of aspergillosis diagnosis	34 (51.5%)	0 (0.0%)	34 (0.0%)	

CSPA = clinically significant pulmonary aspergillosis. * indicates *P* < 0.05

* see Supplementary Table 1 (Data Dictionary) for definitions

**Figure 1.**
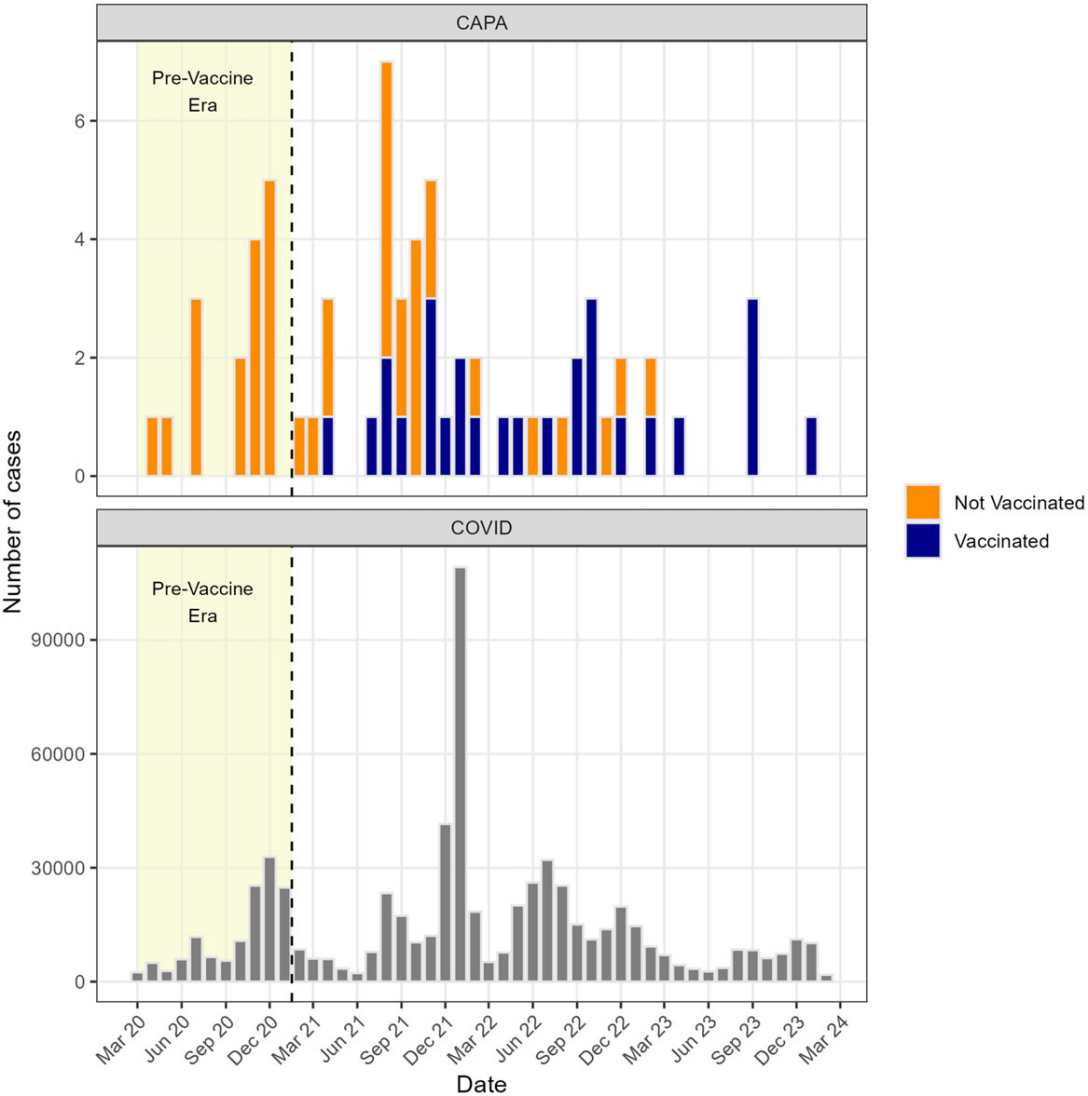
Monthly incidence (total number of cases) of COVID-19 associated pulmonary aspergillosis (CAPA) from March 2020 through March 2024 (top panel) and the monthly incidence of SARS-CoV-2 infection during the same period (bottom panel)—US Department of Veterans Affairs.

The epidemiologic curve of aspergillosis cases is presented in Figure 1. The incidence proportion of aspergillosis was similar in the pre-vaccine (16/124,262; 0.013%) and vaccine (50/550,081; 0.009%) eras. Over time, a growing proportion of patients with post-SARS-CoV-2 aspergillosis had been vaccinated **(**Figure 1). 33/66 (50.0%) patients with aspergillosis died within 90 days after their first positive SARS-CoV-2 test (Table 1). Only 1 patient with aspergillosis who survived more than 90 days after the positive test died within 6 months of the diagnosis of aspergillosis.

**Figure 2.**
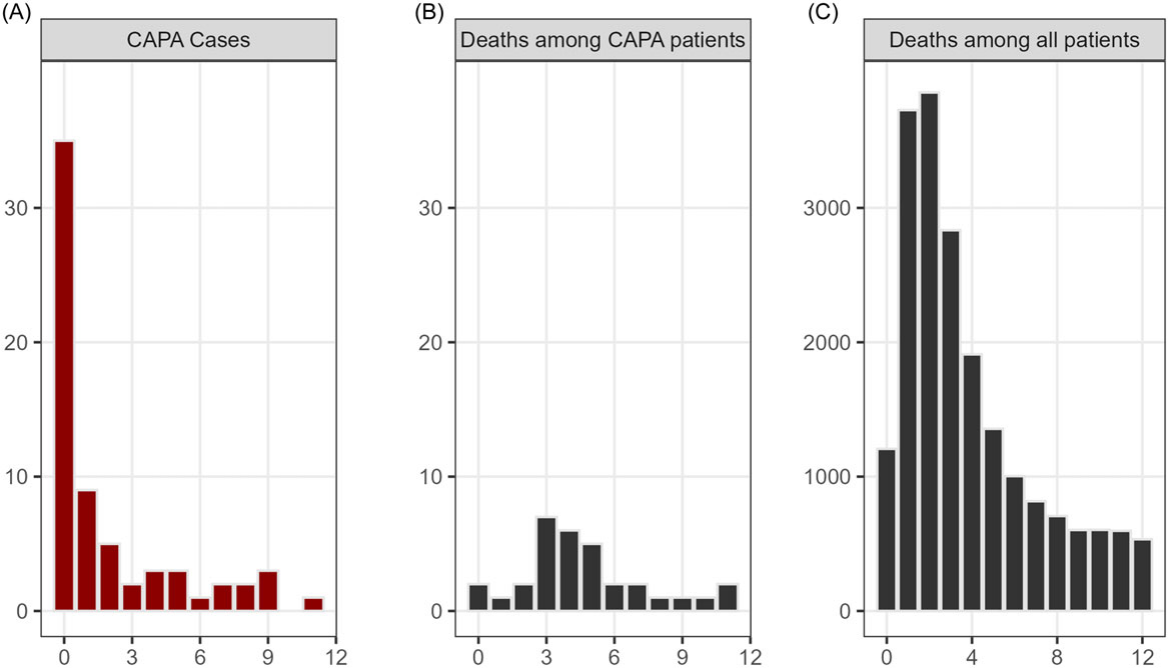
Incidence of clinically significant pulmonary aspergillosis by the number of weeks after the positive test for SARS-CoV-2 (Panel A, only patients with aspergillosis, N = 66), and incidence of death by the number of weeks after the positive test for SARS-CoV-2 among patients with aspergillosis (Panel B) or all patients (Panel C). The data are intended to be descriptive and were not subjected to statistical analysis.

Approximately half (35/66, 53.0%) of cases of aspergillosis were diagnosed within a week of the positive test for SARS-CoV-2 (Figure 2). The timing of mortality among patients with aspergillosis was similar to that among all patients with SARS-CoV-2 infection, or slightly delayed (Figure 2).

**Figure 3.**
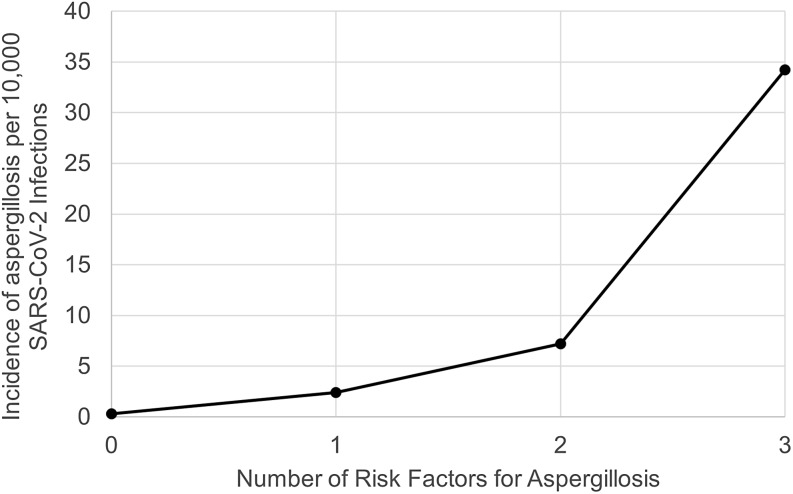
Incidence of Aspergillosis per 10,000 SARS-CoV-2 Infections, by Number of Risk Factors related to compromised immunity (transplantation, prior treatment with immune-compromising drugs, history of hematologic malignancy) or chronic respiratory disease). * Due to small numbers, 3 and 4 risk factors were combined into one category.

Among the 66 patients with aspergillosis, 27 (40.9%) had completed an initial vaccine series, and 52 (78.8%) had at least one traditional risk factor (eg, transplantation, prior treatment with immune-compromising drugs, history of hematologic malignancy, and chronic respiratory disease) for invasive aspergillosis (Table 1). Only 14 (21.2%) of patients with aspergillosis had none of these 4 risk factors. The incidence proportion of aspergillosis following SARS-CoV-2 infection in patients with none of the specified risk factors was 0.3 per 10,000 cases; with one risk factor, 2.4 per 10,000 cases, with two risk factors, 7.2 per 10,000 cases, and among those with three or more risk factors, 34.2 per 10,000 cases (Figure 3). In patients with no specified risk factors who developed aspergillosis, 12/14 (85.7%) had received dexamethasone, compared to 28,477/532,382 (5.3%) who did not develop aspergillosis. Among patients with at least one risk factor, 38/52 (73.1%) of patients with aspergillosis had received dexamethasone, compared to 24,952/141,895 (17.6%) of patients without aspergillosis. Only 2 patients with aspergillosis had no specified risk factors and had not received dexamethasone, and neither of them had been vaccinated.

In the multivariate logistic regression analysis, use of immune-suppressive drugs (aOR 6.47, CI 3.84–10.92), chronic respiratory disease (aOR 3.57, CI 2.10–6.14), and age > 70 (aOR 2.78, CI 1.64–4.80) were all independently associated with increased odds of aspergillosis (Table 1). Infection before (pre-vaccine era) or after (vaccine era) vaccines became available was not associated with odds of aspergillosis (aOR 0.96, CI 0.51–1.85). On an individual basis, vaccination was associated with lower odds of aspergillosis (aOR 0.50, CI 0.28–0.90). Of the 33/66 (50%) patients receiving immune-suppressive drugs prior to the SARS-CoV-2 diagnosis, the majority (24/33, 72.7%; 36.4% of all patients with aspergillosis) were receiving systemic glucocorticoids, either alone (16/24, 66.7%) or in combination with non-glucocorticoids (8/24, 33.3%) (Supplementary Table 2).

## Discussion

In this longitudinal cohort of US Veterans with laboratory-confirmed SARS-CoV-2 infection, we found that the overall incidence of clinically significant pulmonary aspergillosis within the next 12 weeks was quite low, comprising approximately 1 in 10,000 cases, and that incidence proportions before and after vaccines became available were similar. Incidence proportions appeared, descriptively, to be highly correlated with the number of specified risk factors for invasive fungal infections, ranging from 0.3 cases per 10,000 among patients with no risk factors to 34.2 cases per 10,000 among those with three or more risk factors for invasive fungal infections.

Early in the pandemic, prior to widespread population immunity and when risk of severe COVID-19 was higher, post-SARS-CoV-2 aspergillosis cases were identified in patients without traditional risk factors for aspergillosis. In the period after widespread immunity, as cases of severe disease fell in patients without underlying medical conditions, the risk of post-SARS-CoV-2 aspergillosis shifted to comprise a population more likely to be vaccinated. This finding is likely due to the higher rate of vaccination in patients with underlying comorbidities, especially in patients who may not be able to mount a sustained immunologic response to SARS-CoV-2, such as patients receiving immune-suppressive drugs before infection or with hematologic malignancy.

The high use of dexamethasone in patients who developed aspergillosis, also well documented in other studies,^7^ could implicate it as a risk factor for aspergillosis due to immune suppression, as has been previously suggested. However, given the highly colinear relationship between dexamethasone and severe COVID-19, dexamethasone is also a surrogate marker for lung pathology and respiratory failure, which may be the true causal factor. Another possibility is that the combination of immune suppression caused by dexamethasone and the lung pathology caused by severe COVID-19 that together create the potential for an aspergillus infection. In this observational study, we are not able to distinguish between these possibilities. However, post-influenza aspergillus infections are a well described phenomenon that occurs in the absence of receipt of dexamethasone, suggesting that severe underlying lung pathology is a necessary condition for the development of post-viral fungal infections of the lung.

Particularly after vaccines became available, most cases we identified were among patients with conditions associated with risk for pulmonary aspergillosis independent of SARS-CoV-2 infection (immune-suppressive drugs, hematologic malignancy, structural lung disease, transplantation). This same population of patients remain at higher risk of severe disease and resulting lung injury from SARS-CoV-2 and other respiratory virus infections, such as influenza.^19^ These findings suggest that invasive aspergillosis is a general risk among patients with severe respiratory viruses, rather than a risk specific to SARS-CoV-2 infection. Although the populations included are not exactly the same, the rate of post-influenza aspergillosis in another study (0.17%) is higher than was found post-SARS-CoV-2 in our study (0.0098%).^19,20^ Another notable similarity is that risk of post-influenza aspergillosis was higher during the H1N1 outbreak than during periods without novel circulating influenza viruses,^19^ perhaps suggesting that lack of immunity to the virus, resulting in more severe disease and lung damage, is likely a causal factor across multiple respiratory viral diseases. Notably, dexamethasone is not typically used to treat influenza and thus is unlikely to be the driving factor in this population, although prior studies have found that immune suppression from corticosteroids to treat underlying medical conditions is a risk factor, similar to our findings for post-SARS-CoV-2 aspergillosis.^21^

Our study, like others, is limited by how post-SARS-CoV-2 aspergillosis is defined. Given challenges distinguishing colonization from invasive disease, diagnosis of pulmonary aspergillosis should often be questioned—and uncertainty was often conveyed by clinicians in the cases we reviewed. The authors of the consensus definition of CAPA assert that BAL is required, either with a positive culture or galactomannan antigen, to make the diagnosis.^2^ Although this approach would have higher specificity than our method for identifying cases of “clinically significant pulmonary aspergillosis,” BAL was used infrequently in our data set, and applying the strict case definition would have led to identification of only 18 cases. We feel that presenting data on 66 clinician-confirmed cases of aspergillosis following SARS-CoV-2 infection, even if the consensus definition from 2020 was not met, is more valuable than presenting data that would suggest inappropriately that the condition is vanishingly rare in the U.S. Although our definitions are not exactly the same, this approach is congruent with other studies that have used more sensitive definitions and recognized limitations of the strict consensus research definition.^17,18^ We chose research questions about population-level risk, change over time and risk factors with the understanding that we would not be able to provide insight into CAPA and the extent to which it may or may not differ from invasive aspergillosis arising in other settings.

The 3-month mortality among the cases of aspergillosis was high (50%) in this cohort and similar to published estimates.^9,22^ Combined with the fact that only one additional patient who survived 90 days died within 6 months of the diagnosis of aspergillosis, COVID-19 and/or aspergillosis are implicated the cause(s) of or contributor(s) to death, rather than considering aspergillosis to be merely an indicator of underlying poor health. Immune suppression is a traditional risk factor for pulmonary aspergillosis, regardless of SARS-CoV-2 infection, and we are not able to determine if the aspergillosis in this population was attributable to lung injury from COVID-19, the immune-suppressed state, or both.

Although incidence proportions in the post-vaccine era were similar to those in the pre-vaccine era in this study, this finding should be interpreted in light of changes in testing practices over time, which may have caused variation in the denominator of SARS-CoV-2 cases used to calculate the incidence of aspergillosis. Universal testing of inpatients independent of symptoms, as became common throughout the VA, identified many cases of SARS-CoV-2 infection that produced few or no symptoms.^23,24^ Although the calculated incidence proportion of aspergillosis would be lower than during times of more limited use of testing, we cannot estimate the magnitude of the difference, so this study does not allow a strong conclusion that incidence of post-SARS-CoV-2 aspergillosis remained stable over time.

## Limitations

Our study has additional limitations. First, the study was conducted in a VA population. Compared to the general population, the VA population is older, has a much higher percentage of male patients, higher rates of medical co-morbidities, and higher percentages of white and black individuals. Overall, these features would tend to over-estimate the incidence of aspergillosis relative to other populations, as rates of comorbidities are higher. Another limitation of the VA database is that cases of SARS-CoV-2 infection or aspergillosis diagnosed outside of the VA may have been missed. This risk is mitigated by our requirement for SARS-CoV-2 testing to have been performed within the VA, so that the only missed cases of aspergillosis would be patients tested at the VA and then hospitalized elsewhere. Because antifungal treatment for invasive aspergillosis is continued for weeks, and patients make extensive use of the VA pharmacy even when their care is received elsewhere, we propose that few cases of patients who survived aspergillosis were missed. Second, it is likely that some cases were misclassified, particularly cases relying on sputum culture alone as microbiologic support. This approach would overestimate risk, as some cases of colonization would be counted as invasive aspergillus infections. However, the reviewers did not regard sputum culture alone as indicating aspergillosis, but rather studied the reasoning of the clinicians who decided to treat (or not) as invasive aspergillus infection. Third, we did not include all identified risk factors for pulmonary aspergillosis. Due to small numbers, we did not make an effort to evaluate the impact of less commonly used immune suppressive treatments for severe COVID-19, such as baricitinib or tocilizumab,^25^ but the frequent use of dexamethasone was notable even if not clearly interpretable as a risk factor. Fourth, we did not study, neither in the primary analysis nor as a sub-analysis, patients requiring ICU care or mechanical ventilation. However, we regard this as a decision rather than a limitation, once it became clear that the definition of CAPA would be met rarely due to low use of BAL. ICU care is not a perfect correlate of severe respiratory illness among patients with severe comorbidities and a decentralized healthcare system with widely varying local practices and resources, plus many gravely ill patients are not intubated per their own wishes, eg, more patients with aspergillosis in our study died within 90 days (50%) than were intubated (41%).^23^ Finally, we limited the study to the first identified SARS-CoV-2 infection, since distinguishing a new infection from residual virus would require arbitrary decisions about timing and/or a requirement for intervening negative tests.

## Conclusions

In this multi-year, retrospective cohort of US Veteran patients with SARS-CoV-2 infection, we found that the overall incidence proportion of clinically-defined pulmonary aspergillosis is low, affecting approximately 1 in 10,000 confirmed cases of SARS-CoV-2, and that case rates are similar in the pre- and post-vaccination eras. Co-morbidities, particularly underlying immune suppression, are common in identified cases. Among vaccinated patients without traditional risk factors for invasive pulmonary aspergillosis and without use of dexamethasone to treat severe COVID-19, no cases were identified.

## Supporting information

Swinnerton et al. supplementary material Swinnerton et al. supplementary material

## Supplementary material

For supplementary material accompanying this paper visit http://doi.org/10.1017/ash.2024.476. click here to view supplementary material
