# Supplementary material for: Pulmonary aspergillosis in US Veterans with COVID-19: a nationwide, retrospective cohort study
Source: Antimicrob Steward Healthc Epidemiol. 2025 Jan 30;5(1):e28. doi: 10.1017/ash.2024.476 (PMC11795435; doi:10.1017/ash.2024.476)
Supplement: Swinnerton et al. supplementary material [file S2732494X24004765sup001.docx]

**Supplementary Materials**

| **Item** | **Page** |
| --- | --- |
| Supplementary Table 1. Data Dictionary (ICD-10 codes and immunocompromising medications) | 2-6 |
| Supplementary Table 2. Receipt of immunocompromising medications - details | 7 |

**Supplementary Table 1. Data Dictionary.**

**Part A. ICD-10 Codes and Disease Classifications.** % indicates that all sub-headings under the listed code were included. COPD = chronic obstructive pulmonary disease.

| Clinical Disease Group | Disease Phenotype | ICD10_code |
| --- | --- | --- |
| Aspergillosis | Aspergillosis | B44.% |
| Hematalogic malignancies | Hodgkin’s lymphoma | C81.% |
| Hematalogic malignancies | Non-Hodgkin’s Lymphoma | C82.% |
| Hematalogic malignancies | Non-Hodgkin’s Lymphoma | C83.% |
| Hematalogic malignancies | Non-Hodgkin’s Lymphoma | C84.% |
| Hematalogic malignancies | Non-Hodgkin’s Lymphoma | C85.% |
| Hematalogic malignancies | Non-Hodgkin’s Lymphoma | C86.% |
| Hematalogic malignancies | Non-Hodgkin’s Lymphoma | C88.4 |
| Hematalogic malignancies | Non-Hodgkin’s Lymphoma | C91.5% |
| Hematalogic malignancies | Non-Hodgkin’s Lymphoma | C91.A% |
| Hematalogic malignancies | Chronic lymphocytic leukemia | C91.1% |
| Hematalogic malignancies | Chronic lymphocytic leukemia | C91.3% |
| Hematalogic malignancies | Chronic lymphocytic leukemia | C91.4% |
| Hematalogic malignancies | Chronic lymphocytic leukemia | C91.6% |
| Hematalogic malignancies | Acute lymphoblastic leukemia | C91.0% |
| Hematalogic malignancies | Myeloprofilerative disorders | D45. |
| Hematalogic malignancies | Myeloprofilerative disorders | D47.1 |
| Hematalogic malignancies | Myeloprofilerative disorders | D47.3 |
| Hematalogic malignancies | Myeloprofilerative disorders | D47.4 |
| Hematalogic malignancies | Myeloprofilerative disorders | C94.4% |
| Hematalogic malignancies | Myelodysplastic syndromes | C94.6 |
| Hematalogic malignancies | Myelodysplastic syndromes | D46.0 |
| Hematalogic malignancies | Myelodysplastic syndromes | D46.1 |
| Hematalogic malignancies | Myelodysplastic syndromes | D46.2% |
| Hematalogic malignancies | Myelodysplastic syndromes | D46.9 |
| Hematalogic malignancies | Myelodysplastic syndromes | D46.A |
| Hematalogic malignancies | Myelodysplastic syndromes | D46.B |
| Hematalogic malignancies | Myelodysplastic syndromes | D46.C |
| Hematalogic malignancies | Myelodysplastic syndromes | D46.Z |
| Hematalogic malignancies | Myelodysplastic syndromes | C92.2% |
| Hematalogic malignancies | Myelodysplastic syndromes | C93.1% |
| Hematalogic malignancies | Chronic myelogenous leukemia | C93.1% |
| Hematalogic malignancies | Acute myelogenous leukemia | C92.0% |
| Hematalogic malignancies | Acute myelogenous leukemia | C92.4% |
| Hematalogic malignancies | Acute myelogenous leukemia | C92.5% |
| Hematalogic malignancies | Acute myelogenous leukemia | C92.6% |
| Hematalogic malignancies | Acute myelogenous leukemia | C92.A% |
| Hematalogic malignancies | Acute myelogenous leukemia | C93.0% |
| Hematalogic malignancies | Waldenstrom’s macroglobulinemia | C88.0 |
| Hematalogic malignancies | Myeloma and other plasma cell malignancies | C90.% |
| Respiratory disease | Asthma | J45.* |
| Respiratory disease | COPD (chronic bronchitis) | J41.* |
| Respiratory disease | COPD (emphasema) | J43.* |
| Respiratory disease | COPD (with infection) | J44.0 |
| Respiratory disease | COPD (with acute exacerbation) | J44.1 |
| Respiratory disease | COPD (unspecified) | J44.9 |
| Respiratory disease | Bronchiectasis | J47.* |
| Respiratory disease | Chronic bronchitis, unspecified | J42 |
| Respiratory disease | Interstitial pulmonary diseases with fibrosis | J84.1% |
| Transplantation | Kidney transplant | Z94.0 |
| Transplantation | Heart transplant | Z94.1 |
| Transplantation | Lung transplant | Z94.2 |
| Transplantation | Heart and lung transplant | Z94.3 |
| Transplantation | Liver transplant | Z94.4 |
| Transplantation | Bone marrow transplant | Z94.81 |
| Transplantation | Intestine transplant | Z94.82 |
| Transplantation | Pancreas transplant | Z94.83 |
| Transplantation | Stem cell transplant | Z94.84 |

**B. Immunocompromising drugs prescribed before SARS-CoV-2 infection.** Recent use was defined by a maximum number of months since the most recent prescription / administration before SARS-CoV-2 infection, which differed among drug classes. Many drugs used to treat hematologic malignancies are not included, since those conditions were considered separately as a risk factor for aspergillosis.

| Generic | Brand | Category | Maximum months since last use before infection |
| --- | --- | --- | --- |
| arsenic | trisenox | Chemotherapy | 6 |
| asparaginase | asparaginase | Chemotherapy | 6 |
| asparaginase erwinia chrysanthemi | erwinaze | Chemotherapy | 6 |
| azacitidine | vidaza | Chemotherapy | 6 |
| bendamustine | bendeka,treanda | Chemotherapy | 6 |
| bleomycin | bleomycin | Chemotherapy | 6 |
| busulfan | busulfex,myleran | Chemotherapy | 6 |
| cabazitaxel | jevtana | Chemotherapy | 6 |
| calaspargase | asparlas | Chemotherapy | 6 |
| capecitabine | xeloda | Chemotherapy | 6 |
| carboplatin | carboplatin | Chemotherapy | 6 |
| carmustine | bicnu | Chemotherapy | 6 |
| chlorambucil | leukeran | Chemotherapy | 6 |
| chrysanthemi | chrysanthemi | Chemotherapy | 6 |
| cisplatin | cisplatin | Chemotherapy | 6 |
| cladribine | cladribine | Chemotherapy | 6 |
| clofarabine | clolar | Chemotherapy | 6 |
| cyclophosphamide | cyclophosphamide | Chemotherapy | 6 |
| cytarabine | cytosar-u,tarabine pfs | Chemotherapy | 6 |
| dacarbazine | dacarbazine | Chemotherapy | 6 |
| dactinomycin | cosmegen | Chemotherapy | 6 |
| daunorubicin | cerubidine,rubidomycin | Chemotherapy | 6 |
| decitabine | dacogen | Chemotherapy | 6 |
| docetaxel | taxotere | Chemotherapy | 6 |
| doxorubicin | adriamycin | Chemotherapy | 6 |
| epirubicin | ellence | Chemotherapy | 6 |
| eribulin | halaven | Chemotherapy | 6 |
| erwinia | erwinia | Chemotherapy | 6 |
| etoposide | etopophos | Chemotherapy | 6 |
| fludarabine | fludarabine | Chemotherapy | 6 |
| gemcitabine | gemzar | Chemotherapy | 6 |
| hydroxycamptothecin | hydroxycamptothecin | Chemotherapy | 6 |
| hydroxyurea | hydrea | Chemotherapy | 6 |
| idarubicin | idarubicin | Chemotherapy | 6 |
| ifosfamide | ifex | Chemotherapy | 6 |
| irinotecan | camptosar | Chemotherapy | 6 |
| ixabepilone | ixempra | Chemotherapy | 6 |
| lomustine | lomustine | Chemotherapy | 6 |
| mechlorethamine | mustargen | Chemotherapy | 6 |
| melphalan | alkeran for injection,evomela | Chemotherapy | 6 |
| mercaptopurine | purinethol,purixan | Chemotherapy | 6 |
| mitomycin c | mitomycin c | Chemotherapy | 6 |
| mitoxantrone | mitoxantrone | Chemotherapy | 6 |
| nelarabine | arranon | Chemotherapy | 6 |
| oxaliplatin | eloxatin | Chemotherapy | 6 |
| paclitaxel | taxol | Chemotherapy | 6 |
| pegaspargase | oncaspar | Chemotherapy | 6 |
| pemetrexed | alimta | Chemotherapy | 6 |
| pralatrexate | folotyn | Chemotherapy | 6 |
| procarbazine | matulane | Chemotherapy | 6 |
| temozolomide | temodar | Chemotherapy | 6 |
| thioguanine | tabloid | Chemotherapy | 6 |
| thiotepa | thiotepa | Chemotherapy | 6 |
| topotecan | hycamtin | Chemotherapy | 6 |
| trabectedin | yondelis | Chemotherapy | 6 |
| trifluridine | lonsurf | Chemotherapy | 6 |
| valrubicin | valstar | Chemotherapy | 6 |
| vinblastine | vinblastine | Chemotherapy | 6 |
| vincristine | marqibo | Chemotherapy | 6 |
| vindesine | vindesine | Chemotherapy | 6 |
| vinorelbine | navelbine | Chemotherapy | 6 |
| adalimumab | adalimumab | Cytokine-blocking | 3 |
| anakinra | anakinra | Cytokine-blocking | 3 |
| benralizumab | benralizumab | Cytokine-blocking | 3 |
| brodalumab | brodalumab | Cytokine-blocking | 3 |
| canakinumab | canakinumab | Cytokine-blocking | 3 |
| certolizumab | certolizumab | Cytokine-blocking | 3 |
| dupilumab | dupilumab | Cytokine-blocking | 3 |
| etanercept | etanercept | Cytokine-blocking | 3 |
| golimumab | golimumab | Cytokine-blocking | 3 |
| infliximab | infliximab | Cytokine-blocking | 3 |
| ixekizumab | ixekizumab | Cytokine-blocking | 3 |
| mepolizumab | mepolizumab | Cytokine-blocking | 3 |
| rilonacept | rilonacept | Cytokine-blocking | 3 |
| sarilumab | sarilumab | Cytokine-blocking | 3 |
| secukinumab | secukinumab | Cytokine-blocking | 3 |
| tocilizumab | tocilizumab | Cytokine-blocking | 3 |
| ustekinumab | ustekinumab | Cytokine-blocking | 3 |
| methylprednisolone | methylprednisolone | Glucocorticoids | 1 |
| prednisone | prednisone | Glucocorticoids | 1 |
| abatacept | abatacept | Leukocyte-inhibitory | 3 |
| azathioprine | azathioprine | Leukocyte-inhibitory | 3 |
| baricitinib | baricitinib | Leukocyte-inhibitory | 3 |
| belimumab | belimumab | Leukocyte-inhibitory | 3 |
| dimethyl fumarate | dimethyl fumarate | Leukocyte-inhibitory | 3 |
| fingolimod | fingolimod | Leukocyte-inhibitory | 3 |
| leflunomide | leflunomide | Leukocyte-inhibitory | 3 |
| methotrexate | methotrexate | Leukocyte-inhibitory | 3 |
| mycophenolate | mycophenolate | Leukocyte-inhibitory | 3 |
| mycophenolic | mycophenolic | Leukocyte-inhibitory | 3 |
| natalizumab | natalizumab | Leukocyte-inhibitory | 3 |
| rapamycin | rapamycin | Leukocyte-inhibitory | 3 |
| sirolimus | sirolimus | Leukocyte-inhibitory | 3 |
| teriflunomide | teriflunomide | Leukocyte-inhibitory | 3 |
| tofacitinib | tofacitinib | Leukocyte-inhibitory | 3 |
| upadacitinib | upadacitinib | Leukocyte-inhibitory | 3 |
| vedolizumab | vedolizumab | Leukocyte-inhibitory | 3 |
| alemtuzumab | alemtuzumab | Lymphocyte-depleting | 18 |
| obinutuzumab | obinutuzumab | Lymphocyte-depleting | 18 |
| ocrelizumab | ocrelizumab | Lymphocyte-depleting | 18 |
| rituximab | rituximab | Lymphocyte-depleting | 18 |

**Supplementary Table 2**. **Receipt of Immunocompromising Medications in Patients Diagnosed with clinically significant aspergillosis after SARS-CoV-2 infection**. Percentages are shown relative to the number of patients taking one or more immunocompromising drugs (33), which was 50% of all patients with aspergillosis

| N (%) | |
| --- | --- |
| **Overall** | |
| Any immunocompromising drug | 33 (100%) |
| **Drug Category** | |
| Glucocorticoids | 24 (72.7%) |
| Cytokine blocking | 4 (12.1%) |
| Leukocyte inhibitory | 11 (33.3%) |
| Lymphocyte depleting | 2 (6.1%) |
| Chemotherapy | 0 (0%) |
| **Combination Use** | |
| Glucocorticoids only | 16 (48.5%) |
| Non-glucocorticoids only | 9 (27.3%) |
| Glucocorticoids + non-glucocorticoids | 8 (24.2%) |
